# Human iPSCs can be differentiated into notochordal cells that reduce intervertebral disc degeneration in a porcine model

**DOI:** 10.7150/thno.34898

**Published:** 2019-10-12

**Authors:** Dmitriy Sheyn, Shiran Ben-David, Wafa Tawackoli, Zhengwei Zhou, Khosrawdad Salehi, Maxim Bez, Sandra De Mel, Virginia Chan, Joseph Roth, Pablo Avalos, Joseph C. Giaconi, Haneen Yameen, Lena Hazanov, Dror Seliktar, Debiao Li, Dan Gazit, Zulma Gazit

**Affiliations:** 1 Board of Governors Regenerative Medicine Institute, Cedars-Sinai Medical Center, Los Angeles, 90048, CA;; 2 Department of Orthopedics, Cedars-Sinai Medical Center, Los Angeles, 90048, CA;; 3 Department of Surgery, Cedars-Sinai Medical Center, Los Angeles, 90048, CA;; 4 Department of Biomedical Sciences, Cedars-Sinai Medical Center, Los Angeles, 90048, CA;; 5 Biomedical Research Imaging Institute, Cedars-Sinai Medical Center, Los Angeles, 90048, CA;; 6 Skeletal Biotech Laboratory, Hebrew University of Jerusalem, 91120, Israel; 7 Faculty of Biomedical Engineering, Technion, Haifa, 32003, Israel

**Keywords:** Differentiation, human iPSCs, notochordal cell phenotype

## Abstract

**Introduction**: As many as 80% of the adult population experience back pain at some point in their lifetimes. Previous studies have indicated a link between back pain and intervertebral disc (IVD) degeneration. Despite decades of research, there is an urgent need for robust stem cell therapy targeting underlying causes rather than symptoms. It has been proposed that notochordal cells (NCs) appear to be the ideal cell type to regenerate the IVD: these cells disappear in humans as they mature, are replaced by nucleus pulposus (NP) cells, and their disappearance correlates with the initiation of degeneration of the disc. Human NCs are in short supply, thus here aimed for generation of notochordal-like cells from induced pluripotent cells (iPSCs).

**Methods**: Human iPSCs were generated from normal dermal fibroblasts by transfecting plasmids encoding for six factors: OCT4, SOX2, KLF4, L-MYC, LIN28, and p53 shRNA. Then the iPSCs were treated with GSK3i to induce differentiation towards Primitive Streak Mesoderm (PSM). The differentiation was confirmed by qRT-PCR and immunofluorescence. PSM cells were transfected with Brachyury (Br)-encoding plasmid and the cells were encapsulated in Tetronic-tetraacrylate-fibrinogen (TF) hydrogel that mimics the NP environment (G'=1kPa), cultured in hypoxic conditions (2% O2) and with specifically defined growth media. The cells were also tested *in vivo* in a large animal model. IVD degeneration was induced after an annular puncture in pigs, 4 weeks later the cells were injected and IVDs were analyzed at 12 weeks after the injury using MRI, gene expression analysis and histology.

**Results**: After short-term exposure of iPSCs to GSK3i there was a significant change in cell morphology, Primitive Streak Mesoderm (PSM) markers (Brachyury, MIXL1, FOXF1) were upregulated and markers of pluripotency (Nanog, Oct4, Sox2) were downregulated, both compared to the control group. PSM cells nucleofected with Br (PSM-Br) cultured in TF hydrogels retained the NC phenotype consistently for up to 8 weeks, as seen in the gene expression analysis. PSM-Br cells were co-cultured with bone marrow (BM)-derived mesenchymal stem cells (MSCs) which, with time, expressed the NC markers in higher levels, however the levels of expression in BM-MSCs alone did not change. Higher expression of NC and NP marker genes in human BM-MSCs was found to be induced by iNC-condition media (iNC-CM) than porcine NC-CM. The annular puncture induced IVD degeneration as early as 2 weeks after the procedure. The injected iNCs were detected in the degenerated discs after 8 weeks *in vivo*. The iNC-treated discs were found protected from degeneration. This was evident in histological analysis and changes in the pH levels, indicative of degeneration state of the discs, observed using qCEST MRI. Immunofluorescence stains show that their phenotype was consistent with the *in vitro* study, namely they still expressed the notochordal markers Keratin 18, Keratin 19, Noto and Brachyury.

**Conclusion**: In the present study, we report a stepwise differentiation method to generate notochordal cells from human iPSCs. These cells not only demonstrate a sustainable notochordal cell phenotype *in vitro* and *in vivo*, but also show the functionality of notochordal cells and have protective effect in case of induced disc degeneration and prevent the change in the pH level of the injected IVDs. The mechanism of this effect could be suggested via the paracrine effect on resident cells, as it was shown in the *in vitro* studies with MSCs.

## Introduction

Low back pain (LBP) is one of the most frequent causes of morbidity and disability 1. It occurs most often between the ages of 30 and 50 years, due in part to the aging process but also as a result of genetic predisposition, mechanical injury, and a sedentary life style 2, 3. As many as 80% of adults experience LBP at some point in their lifetimes 4. The resulting impact of lost productivity, dispersal of disability benefits, and medical and insurance costs is estimated to total $50-$200 billion annually 5. Imaging studies have indicated a link between LBP and intervertebral disc (IVD) degeneration in 40% of patients 6. IVD degeneration is believed to be the major source of chronic back pain, and more than 90% of surgical spine procedures are performed because of consequences of this degenerative process 7. Cells occupy only 1% of IVD volume, but they are responsible for extracellular matrix synthesis and degradation. In disc degeneration, there is a decrease in cell numbers and altered cell function in the nucleus pulposus (NP), resulting in an imbalance between matrix synthesis and degradation. Proteoglycan synthesis decreases, and there is a transition from type II collagen to type I collagen 8. This results in a more fibrous, dehydrated matrix that is less able to withstand the mechanical forces applied to the spine. These changes have been linked to the initiation of pain responses, either through direct ingrowth of nerves and blood vessels into the IVD 9 or by surrounding anatomical structures that put constant pressure on the IVD (e.g., spinal facets, spinal muscles), which further impair IVD function and lead to pain. Current treatments for degenerative disc disease rely on pain management and exercise; when unsuccessful, surgery is indicated. Surgical treatments such as spinal fusion and disc replacement provide satisfactory results in alleviating pain but are not devoid of complications and poor long-term clinical outcomes 10-12. Despite decades of research, robust therapies targeting underlying causes, rather than symptoms, remain in the earliest stages of development 13-17. Thus, there is an urgent need for an alternative treatment such as stem cell therapy, which is focused on correcting the underlying pathogenesis of degenerative disc disease.

Although an optimal cell source remains elusive, a few studies have demonstrated the therapeutic effect of mesenchymal stem cell (MSC) injection into the IVD 18, 19. MSCs have been found either to obtain the NP cell phenotype 20, thereby potentially only serving as a short-term solution, or to induce mineralization and bone tissue formation in the injured IVD 21, which impairs its function. Several studies involving animal models of disc degeneration have demonstrated that transplantation of IVD-derived cells, specifically NP cells, delays the progressive degenerative process and, in some cases, promotes disc regeneration in animal models of IVD degeneration 22. However, these cells were derived from normal NP tissue, which is impossible to obtain without damaging the IVD.

The NP is formed from the embryonic notochord as it segments during fetal development; the surrounding annulus fibrosus (AF) is formed from the mesoderm/sclerotome. At the time of birth, the NP is populated by morphologically distinct, large vacuolated notochordal cells (NCs). In some vertebrates these NCs persist throughout most of adult life, whereas in other species, including humans, NCs gradually disappear during maturation 23, eventually becoming undetectable. They are replaced by a population of smaller round cells, NP cells, which are believed to differentiate from the NCs 24. This change in cell population correlates with the initiation of degenerative changes within the disc, suggesting that the loss of NCs may be responsible for disc degeneration. Interestingly, animals in which NCs remain throughout the majority of their lifespan, including commonly used experimental animals such as rabbits, rats, mice and pigs, do not exhibit signs of spontaneous disc degeneration and maintain a more hydrated proteoglycan-rich matrix than that found in adult human NP tissue 25. Therefore, in this study we used injury-induced porcine disc degeneration model. Although the pigs retain their notochordal cells, but the injury causes collapse of the disc and resident notochordal cell do not suffice for regeneration 26, 27. Supporting this theory, NCs have been found to be more metabolically active and to produce more proteoglycans than smaller NP cells 28. Additionally, the results of *in vitro* experiments with human 29 and bovine 30, 31 NP cells encapsulated in three-dimensional (3D) hydrogels suggest that NCs could also act as stimulators, controlling the synthesis of proteoglycans by NP cells. We can infer from these findings that the development of stem cell-based therapies focusing on differentiation toward an NC phenotype capable of synthesizing a proteoglycan-rich matrix and playing a protective role in a catabolic environment 32 may be more desirable than therapies focusing on treatments based on stem cell differentiation into NP cells.

Given the aforementioned evidence, NCs appear to be ideal cells with which to regenerate the NP. Unfortunately, human NCs are in short supply, due to their disappearance during childhood, and cannot be harvested as an autologous or allogeneic graft. An alternative strategy would be to mimic the differentiation process that occurs during embryogenesis and obtain NCs from pluripotent stem cells. Induced pluripotent stem cells (iPSCs) can be generated today from almost any type of somatic cell by using an integration-free method. The unlimited proliferation capacity of iPSCs, combined with their pluripotent differentiation potential, places them among the most promising stem cells for IVD therapy. Although no iPSCs are used clinically yet, the field of induced pluripotency has been growing rapidly in the last years 33. Because of these cells' fast growth and high plasticity, direct transplantation of iPSCs can result in teratoma formation *in vivo*; however, this capacity of iPSCs can be avoided by pre-differentiation into committed cell lineages 34. iPSCs can be obtained as autologous stem cells reprogrammed from the somatic cells of the patient or as allogeneic cells. We speculate that an allogeneic cell strategy will be more attractive since the IVD is considered immunoprivileged and HLA matching repositories are being established all over the world. Several previous studies attempted to differentiation of pluripotent stem cells using either non-chemically defined NP matrix as the main differentiation agent 35, 36 or using mouse cells 37-39. The Setton group recently published a groundbreaking study in which iPSCs were reprogrammed directly to NP cells 40, but these cells were not tested *in vivo*. In the present study, we report efficient differentiation of human fibroblast-derived iPSCs into NCs, and we describe the survival and stable phenotype of these NCs *in vitro* and *in vivo* in an NP-like environment in a large animal model of IVD degeneration.

The origin of the notochordal cells is not fully defined, however there are some evidence that they develop from the Primitive Streak Mesoderm during embryogenesis 41, 42. Cell differentiation was performed in a stepwise developmental method to generate primitive streak mesoderm (PSM) cells, the initial step in notochord development, notochordal progenitor cells, and subsequently, mature NCs from iPSCs, using overexpression of T-Box Brachyury transcription factor, which has been shown to mediate cartilage development 43. In addition to testing these iPSC-derived NCs (iNCs) survival in an NP-like environment for 8 weeks *in vitro*, we also tested their sustainability *in vivo* in a previously published large animal model of IVD degeneration 26, 27. Recent advances in MRI technologies allow researchers to noninvasively assess changes in pH within the body. Of particular note, chemical exchange saturation transfer (CEST) has been studied to measure pH-dependent signal changes 44, 45. This technique exploits the chemical exchange, which is pH sensitive, between water protons and solute protons in certain molecules. Previous studies have applied CEST to detect pH changes in the IVDs of pigs 27, 46 and human patients 47. We applied this method in studies focused on the *in vivo* model in order to track pH changes correlated with disc degeneration.

## Results

**Figure 1:**
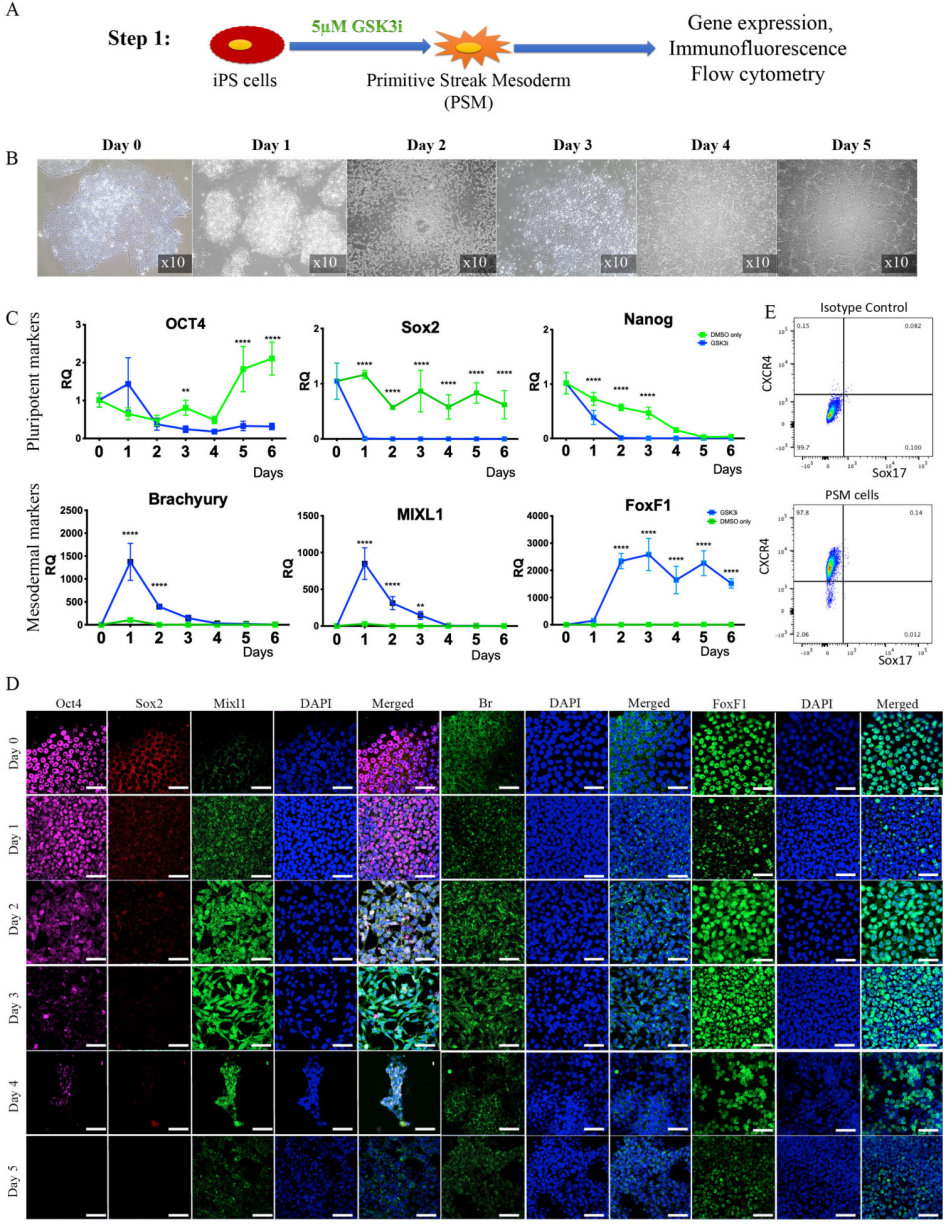
** Step 1 - Differentiation of iPSCs into primitive streak mesoderm (PSM) cells. (A)** Schematic representation of Step 1: iPSCs were treated with GSK3i and tested daily for expression of markers. **(B)** Morphological changes during GSK3i treatment. **(C)** Gene expression analysis of PSM cells shows a rapid decline in the expression of pluripotency markers (Nanog, Oct4, and Sox2) and an increase in mesodermal markers (MIXL1, BR, and FoxF1) in GSK3i-treated cells when compared to DMSO-treated cells. Results were calibrated relative to iPSCs (Day 0). Values are expressed as means ± standard errors (bars); n=6, *p<0.005; **p<0.01; ***p<0.001; ****p<0.0001. **(D)** Immunofluorescence staining of PSM cells for pluripotent and mesodermal markers was performed daily. The highest expression of mesodermal markers (Br, Mixl1, and FoxF1) was observed on Day 3 in conjunction with decreased expression of pluripotent markers (Oct4 and Sox2). **(E)** Flow cytometry of PSM cells for mesodermal CXCR4 marker and endoderm marker Sox17^48^, showing that the PSM cells are homogeneous population of cells that differentiates exclusively to mesoderm.

***Step 1: Differentiation of iPSCs into primitive streak mesoderm (PSM) cells in vitro.*** Human iPSCs were treated with GSK3i (Fig. 1A) and significant morphological changes were observed in the cells every 24 hours (Fig. 1B). Twenty-four hours after treatment commenced, the cells lost their monolayer morphology and appeared cloudy. On Day 2 post treatment, this cloudiness disappeared, and the cells attained a fibroblast-like morphology and dispersed throughout the tissue culture plate in a monolayer. On Day 3, we observed a significant proliferation of cells until they reached full confluence. During the following days, we witnessed a decline in cell abundance and major cell death. The gene expression of pluripotency markers Nanog, Oct4, and Sox2 rapidly declined, as compared to cells cultured in the same Advanced RPMI medium with addition of DMSO, the GSK3i carrier (Fig. 1C).

Immunofluorescent staining of the cells, which was performed daily, confirmed decreases in the Sox2 and Oct4 pluripotency markers that became nearly undetectable on Day 3; concurrently, high expression of the primitive streak mesoderm markers Brachyury and Mixl1 48 was noted (Fig. 1D). Interestingly, increase in expression of Brachyury and Mixl1 was apparent 24 hours after GSK3i treatment; these markers' expression slowly declined, as shown by qRT-PCR, although immunofluorescence staining demonstrated continued high expression on Day 3 (Fig 1C & D). Surprisingly, no Noto expression was detected in the PSM cells (data not shown) at this step, though it was detected in the next step of differentiation.

Based on the observations described above, we chose Day 3 as the most appropriate time to define the state of cell differentiation as primitive streak mesoderm (PSM) cells, and we therefore used cells at Day 3 for the next step. To confirm the exclusive mesodermal differentiation and homogeneity of the PSM population, the cells were tested for surface markers expression associated with mesoderm (CXCR4) and endoderm (Sox17)^48^. The flow cytometry clearly shows that the PSM cells are homogenic in their mesodermal profile and no off-target differentiation to endoderm was detected (Fig. 1E).

**Figure 2:**
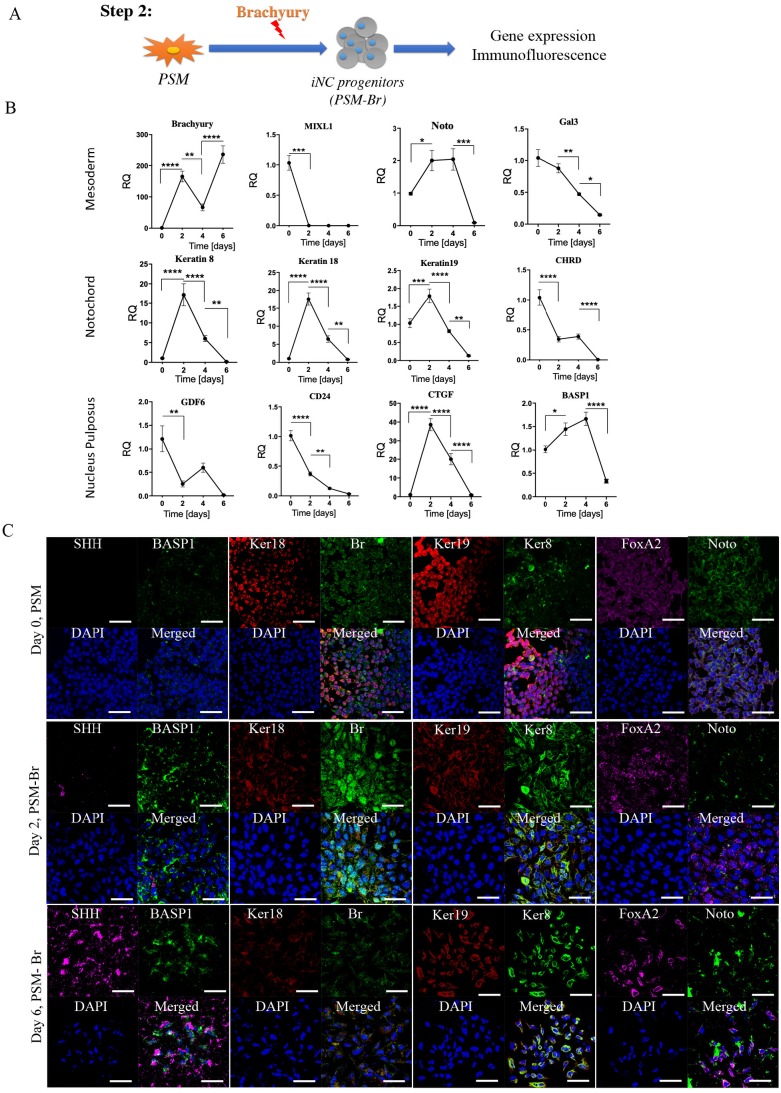
** Step 2 - Differentiation of PSM cells into iNC progenitors**. **(A)** Schematic representation of Step 2: PSM cells were transfected with Br-encoding plasmid and placed in 2D culture.** (B)** On Days 2, 4, and 6 gene expression analyses were performed. They showed a rapid reduction in notochordal markers in both groups, but an elevation in the transfected gene over time. Values are expressed as means ± standard errors (bars); n=6, *p<0.05; **p<0.01; ***p<0.001; ****p<0.0001.** (C)** Immunofluorescence staining was performed for the NC markers Br and Keratin 8 (green), BASP1 and Keratin 18 (red), and SHH and FoxA2 (pink). The analyses confirmed the highest expression of NC markers on Day 2. We concluded that after 2 days *in vitro* NC progenitors start to lose their phenotype, and consequently we used Day 2 cells for our further experiments.

***Step 2: Differentiation of PSM cells into iNC progenitors (PSM-Br).***To further differentiate PSM cells towards iPSC-derived notochordal progenitor cells (iNC progenitors), we induced overexpression of one of the main transcription factors involved in notochord development, Brachyury, by using a nonviral transfection system based on nucleofector technology 49, 50. The nucleofected cells were seeded *in vitro* and their gene expression was analyzed using qRT-PCR and by immunofluorescence staining proteins were detected. The gene expression analysis showed that 2 days after nucleofection with Brachyury-encoding plasmid, the primitive streak mesoderm marker Mixl1 was rapidly downregulated (Fig. 2B). Notochordal markers Keratin 8, Keratin 18, Keratin 19, CD24, Gal3, and CTGF 51, on the other hand, were upregulated on Day 2 after transfection, but this upregulation rapidly declined after 4 and 6 days *in vitro*. Some markers displayed peak expression on Day 4, namely Noto, BASP1, CHRD, and GDF6; however, this expression was not significantly higher than that recorded on Day 2. Immunofluorescence analysis of the cells *in vitro* also showed higher expression of notochordal markers on Day 2 followed by a decline in expression on Day 6 (Fig. 2C), especially with respect to Keratin 8, Keratin 18, BASP1, and FoxA2, but with increased expression of SHH. Taking all these results into account, we concluded that following Brachyury overexpression, if the cells have been cultured in two-dimensional (2D) culture conditions, rapidly lose their phenotype; thus, we chose to continue to the next step: maturation of iNC progenitors in an NP-like environment using PSM overexpressing Brachyury (PSM-Br) Day 2 cells.

**Figure 3:**
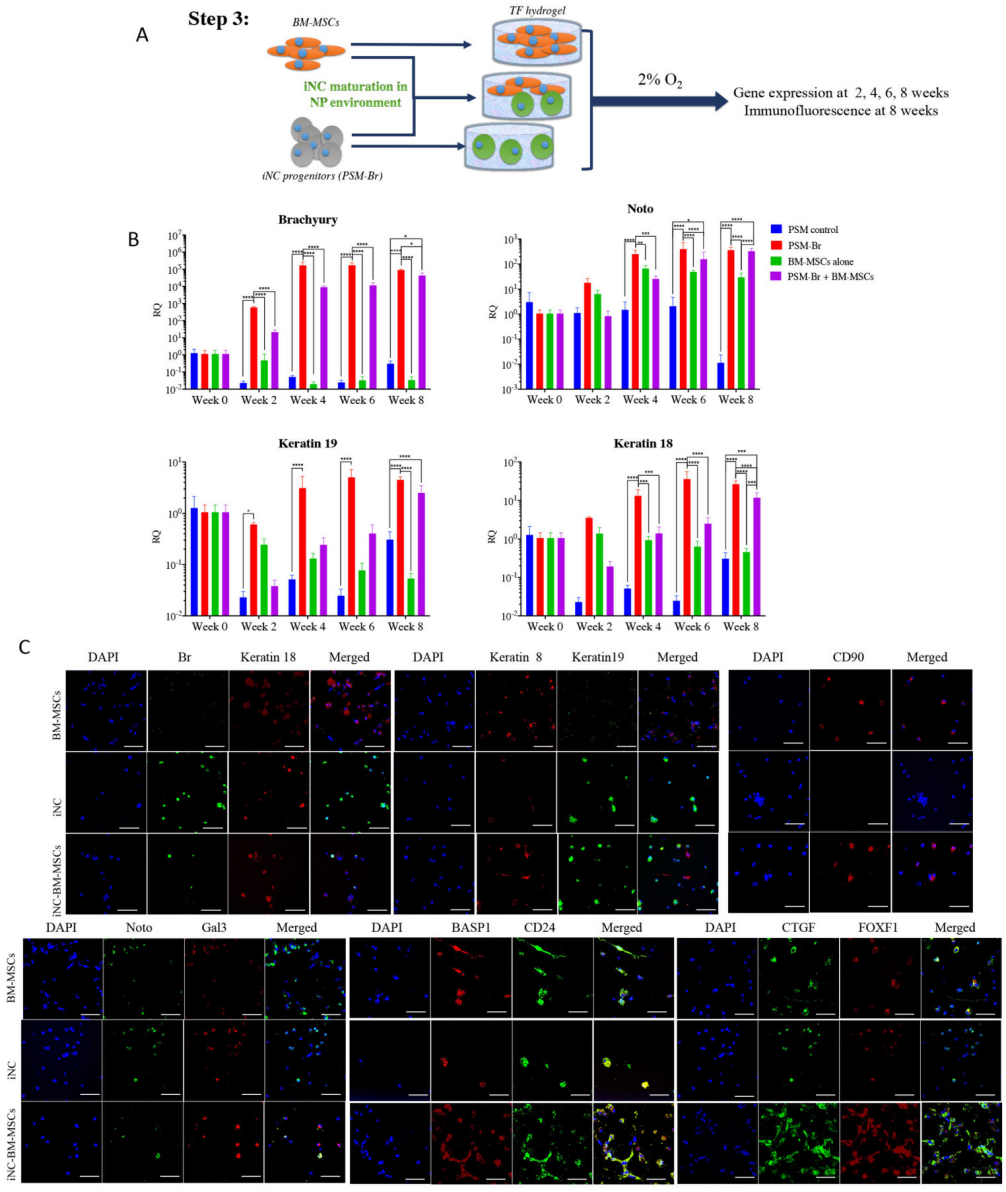
** Step 3 - Maturation of iNC progenitors into iNCs in an NP-like environment and a paracrine effect on BM-MSCs. (A)** PSM-Br Day 2 cells were embedded in TF gel and cultured in 2% O_2_ and NP media for up to 8 weeks. Additionally, the iNCs were mixed with BM-MSCs and co-cultured in TF gels in order to test the paracrine effect of iNCs on BM-MSCs. As a control, BM-MSCs were cultured alone in the same settings. **(B)** Every 2 weeks, TF gels were extracted and tested for gene expression of notochordal markers (Brachyury, Noto, Keratin 19 and Keratin 18) using qRT-PCR. Results show retention of the NC phenotype of the PSM-Br cells once cultured in the NP-like environment. Results are presented as mean RQs calibrated to PSM cells (Day 0). Values are expressed as means ± standard errors (bars); n=6, *p<0.05; **p<0.01; ****p<0.0001. **(C)** Immunofluorescence staining was performed for NC markers (Br, Keratins 8, 18 and 19, Noto, Gal3, and BASP1)^51^, ^65^, one of the MSC markers (CD90)^53^ and NP markers (FOXF1, CTGF and CD24)^51^, ^97^ at the end point of the study (8 weeks). NP and MSC markers were expressed in BM-MSC-included constructs and less so in iNCs only constructs.

***Step 3: Maturation of iNC progenitors into notochordal cells and their long-term culture in a simulated NP environment.***To further differentiate iNC progenitors, the PSM-Br Day 2 cells, bone marrow MSCs (BM-MSCs), or a mixture of both types of cells in a 1:1 ratio were aliquoted and encapsulated in Tetronic1307-Fibrinogen (TF) hydrogels (Fig. 3A) as previously described 52. The gels were cultured in NP-specific medium in hypoxic conditions 37. The hydrogel constructs were harvested on Weeks 4, 6, and 8 for marker gene expression analysis. Expression of the main notochordal markers, Brachyury, Noto, Keratin 18, and Keratin 19, was low in constructs containing only BM-MSCs, stable in constructs containing iNCs, and slightly elevated from one time point to the next in constructs containing a mixed population of cells (Fig. 3B). Immunofluorescence staining of cells at Week 8 revealed CD90, a recognized MSC marker expression 53 in constructs containing MSCs but not in constructs containing only iNCs. CTGF and FoxF1, mainly expressed in NP cells, were expressed in higher levels in constructs containing both cell types, suggesting differentiation of MSCs into the NP phenotype. The notochordal markers Br, Keratins 18 and 19, Noto, and Gal3 were expressed in the construct containing only iNCs as well as in the construct containing both iNCs and MSCs (Fig. 3C). After 8 weeks in a 3D culture in hypoxic conditions, MSC-containing constructs showed positive expression of Basp1, CD24, Keratin 8, CTFG, and FOXF1, suggesting a “nucleus pulposus” cell phenotype.

### Functional analysis of iNC progenitors' conditioned media on human BM-MSCs

**Figure 4:**
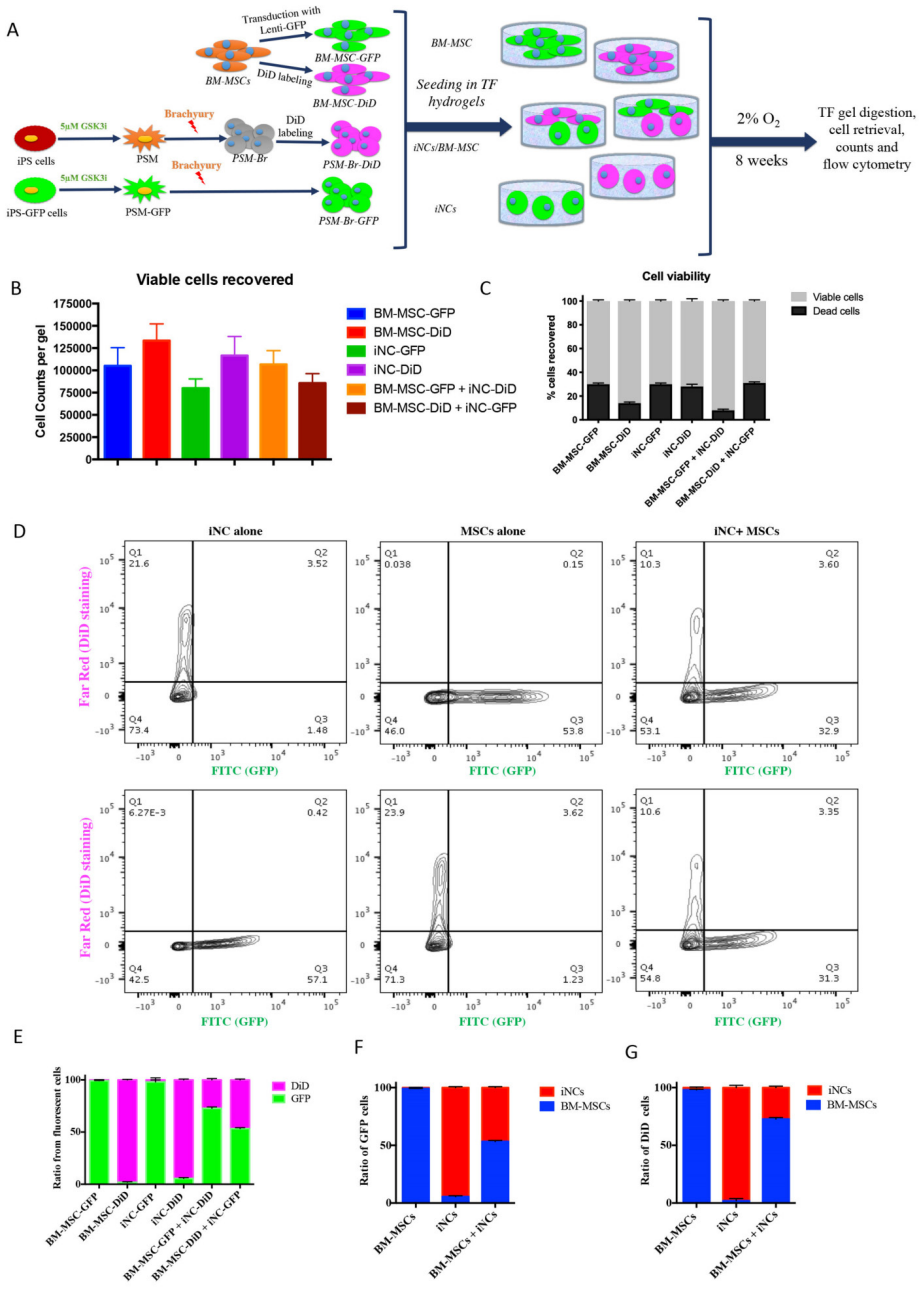
** Survival of iNC + BM-MSC cocultures in TF constructs.** iNC and BM-MSC were stained with DiD lipophilic dye and encapsulated in TF gels alone or with iNC-GFP or BM-MSC-GFP according to the diagram **(A)**. The constructs were cultured in hypoxic NP conditions for 8 weeks, gels digested, the cells recovered and counted using Countess™ to assess viability **(B, C)**. Flow cytometry plots show distribution of GFP expressing cells (green) or DiD-labeled cells (pink) **(D).** Quantitative analysis of separate constructs shows ratio between the green and pink cells **(E)** or when separated and analyzed based on GFP expressing cells **(F)** or DiD-labeled cells **(G)**. Values are expressed as means ± standard errors (bars), n=6.

The elevated expression of notochordal markers in the mixed cell population may have been caused by a paracrine effect of iNCs on MSCs in the NP-mimicking environment. Alternatively, survival of iNCs may be greater than that of MSCs in NP conditions, and thus the greater contribution of iNCs to the overall RNA pool may explain this phenomenon. To test either of these options, an additional experiment was designed in which iPSC and BM-MSCs were labeled with GFP reporter gene under constitutive promoter using lentiviral vector 54, 55. To distinguish between the transgenic GFP cells and non-GFP cells, the later were labeled with lipophilic DiD dye. iNC-DiD and iNC-GFP were derived using the same 3-step protocol and cultured in TF gels, either alone or cocultured with BM-MSC-GFP or BM-MSC-DiD respectively (Fig. 4A). After 8 weeks, the gels were digested with collagenase as previously described 56, the cells were recovered, counted, their viability assessed and analyzed using flow cytometry. In all constructs, very similar number of viable cells were recovered, maintaining close to the original number of seeding, 10^5^ cells per gel (Fig. 4B). No statistical differences in the total number of cells (viable and non-viable) were found between the different cell groups (Fig 4C). Flow cytometry of the cells recovered from the gels showed, as expected, that the gels containing one cell type resulted in only one fluorescent signal, whereas in the gels with both cell types the ratio between iNCs and MSCs remained either the same (50:50) for iNC-GFP + BM-MSC-DiD, or shifted toward MSCs (70:30) when iNC-DiD and BM-MSC-GFP were co-cultured (Fig. 4D-G). It is worthy to note that no events were registered in the double labelling green/red quadrant of the plot, clearly indicating that the cells in co-culture didn't fuse or clump, rather maintained as single cells. In any case, these results confirmed that the shift in gene expression of the co-cultured cells in the previous experiment indicates paracrine effect of the iNCs on MSCs and not poor survival of the MSCs in hypoxic conditions.

**Figure 5:**
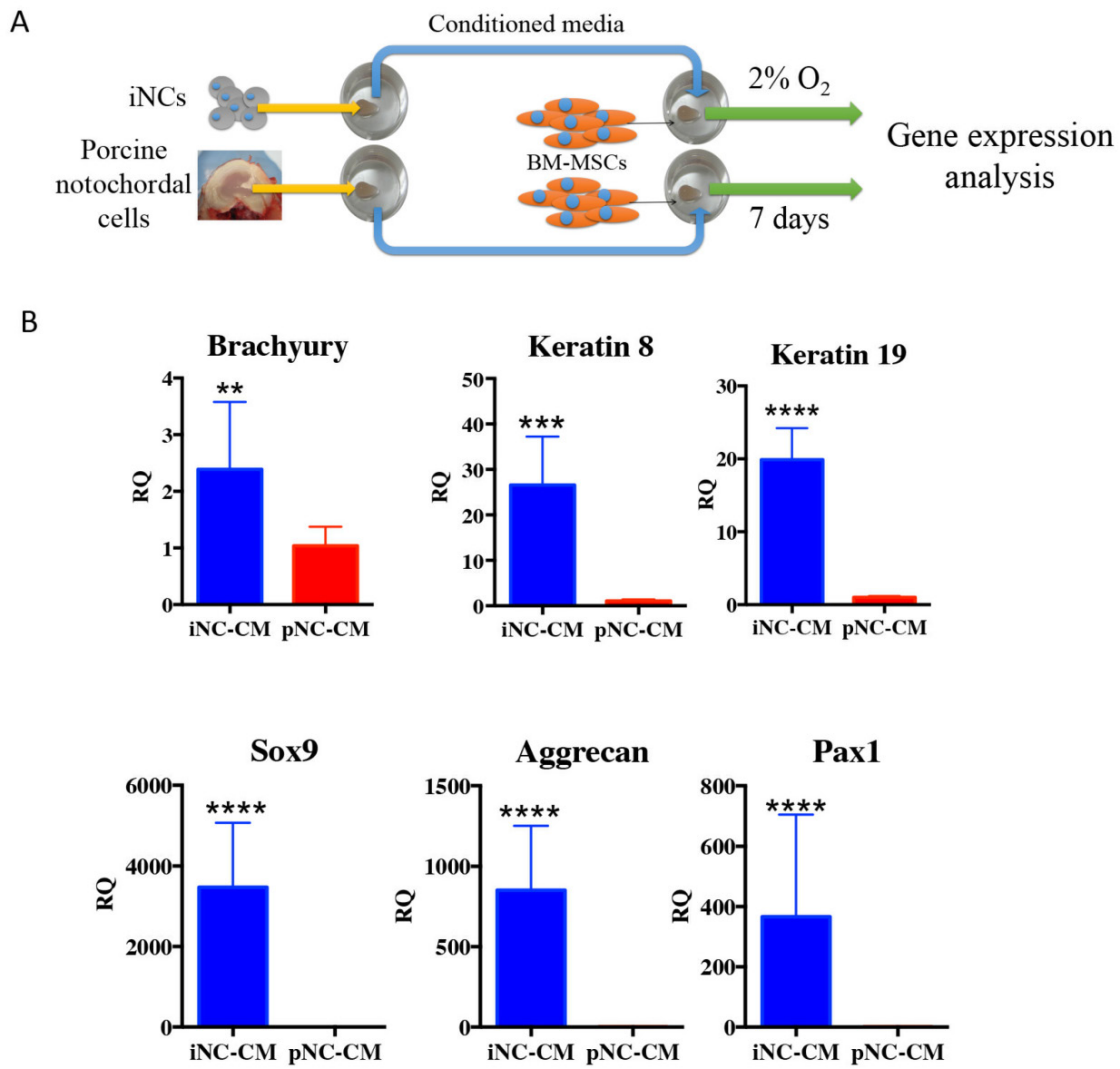
** iNC functional assay: paracrine effect on BM-MSC.** Conditioned medium (CM) was collected from iNCs or primary porcine NCs (pNC) grown in alginate beads. BM-MSCs in alginate beads were grown in either iNC-derived conditioned medium (iNC-CM) or in porcine NC-derived conditioned media (pNC-CM) and analyzed on Day 7. Values are relative to BM-MSCs grown in regular media. Values are expressed as means ± standard errors (bars), n=5; *p<0.05; ***p<0.001; ****p<0.0001.

The outcome of the paracrine effect of iNCs was examined by a gene expression analysis of BM-MSCs after 1 week of culture in porcine NC-derived conditioned medium (pNC-CM) or iNC-conditioned medium (iNC-CM). The results showed higher expression of NP markers in BM-MSCs cultured with iNC-CM comparing to BM-MSCs cultured with pNC-CM (Fig. 5B). Significant upregulation of Keratin 8, Keratin 19, Sox9, Aggrecan, and Pax1 was observed in BM-MSCs after culture in iNC-CM. These results indicate that the paracrine effect of iNC-CM on BM-MSCs exceeds the effect of pNC-CM. Although the comparison of porcine NC-conditioned medium to human iNC-derived conditioned medium is not ideal due to the fact that activity of porcine factors secreted by the cells might be different from human factors. However, the proper comparison using human NC cells was not practical due to unavailability of those cells. Furthermore, the activity and the effect of the animal NCs on human MSCs was demonstrated numerous previous studies 57-59.

### Teratoma formation assay *in vivo*

PSM cells and PSM-Br cells were prepared in the same manner described earlier (Step 1 and Step 2, respectively) and stained with CM-DiI for future tracking. One million cells were injected intramuscularly into immunocompromised NOD/SCID mice (n = 10) as previously described 60, 61. The mice were followed up weekly for 6 months, and no tumor or teratoma formation was observed in any mice injected with the any of the iPSC-derived cells.

### Survival and phenotype retention of iNCs in modeled porcine degenerated disc

**Figure 6:**
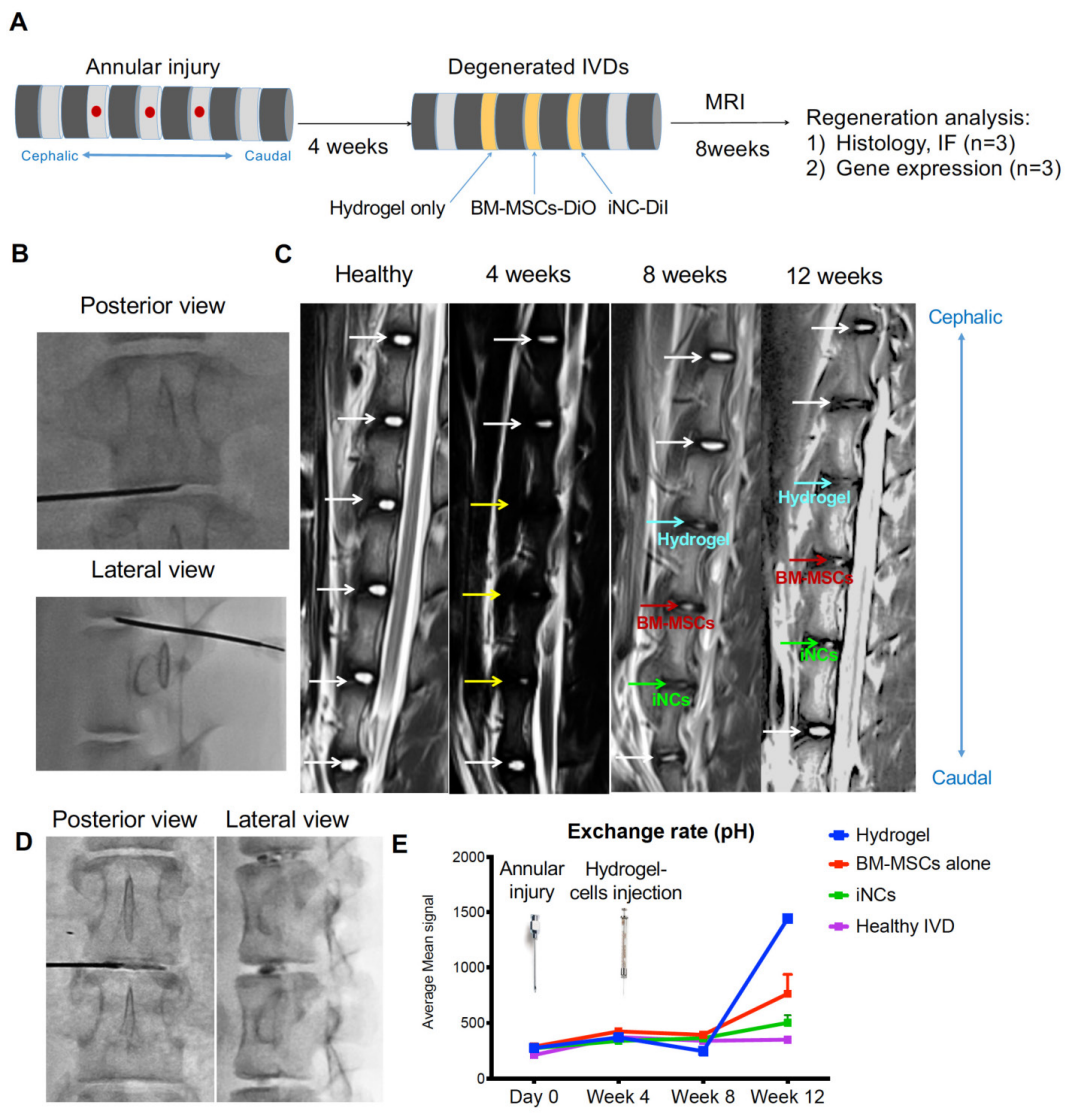
** Porcine IVD degenerated and injected with iNCs: imaging analysis. (A)** Schematic diagram for the experimental design.** (B)** Three levels of IVDs were subjected to annular puncture with a 14G needle under fluoroscopic guidance. **(C)** The degeneration process was imaged using MRI. The first image was obtained before induction of degeneration and shows all healthy IVDs. Subsequent images were taken 4, 8, or 12 weeks after annular puncture. **Yellow arrows** indicate injured IVDs and **white arrows** indicate healthy IVDs.** (D)** 4 weeks after induction of degeneration, DiI-labeled iNCs, BM-MSCs, or hydrogel alone were injected into the NP. **(E)** qCEST imaging that was previously correlated to the pH measured inside the disc. Values are expressed as means ± standard errors (bars), n=3; ****p<0.0001 (comparing to hydrogel only).

**Figure 7:**
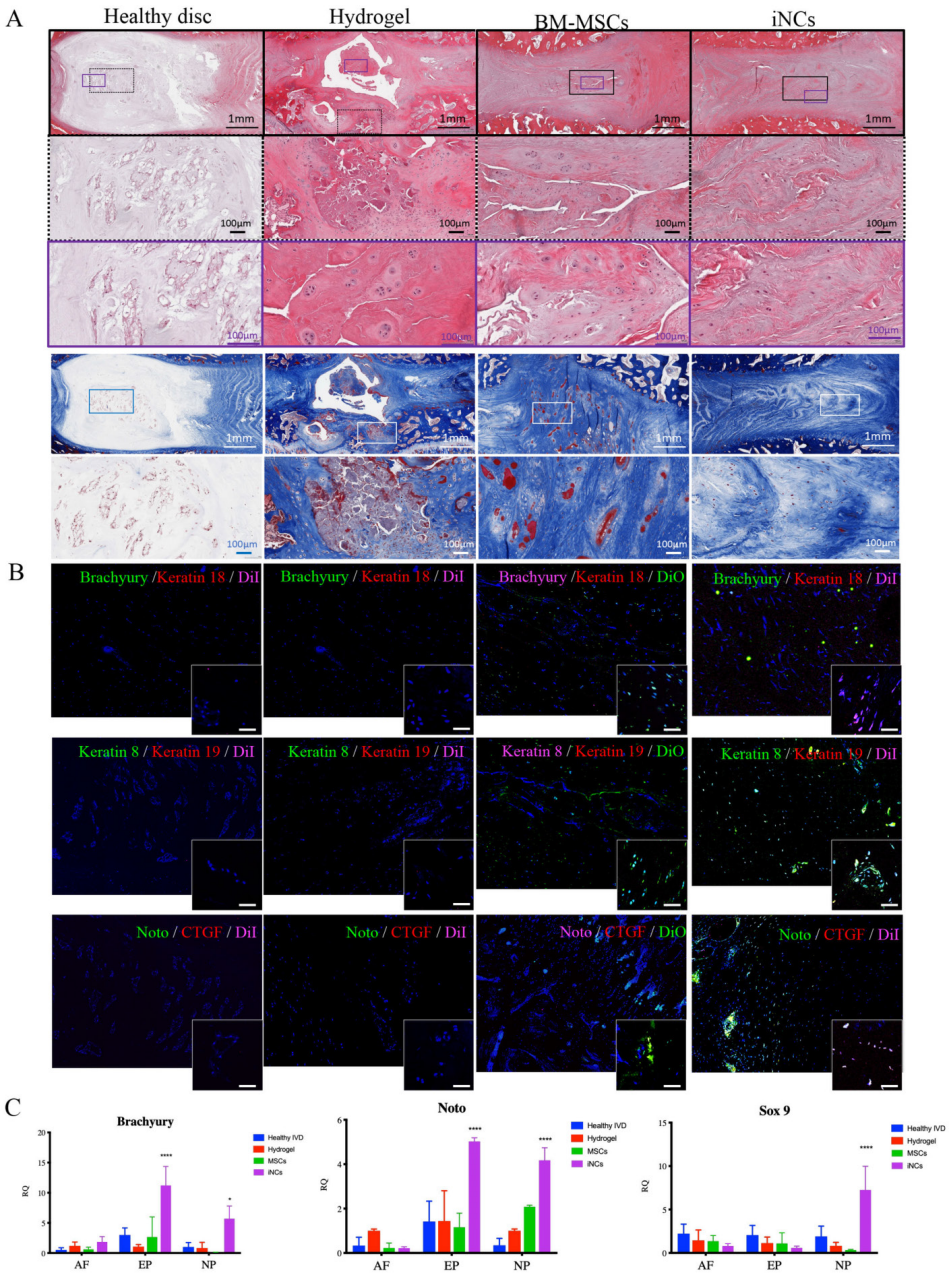
** iNCs survive and maintain their phenotype 8 weeks after intradiscal injection.** The IVDs were harvested 8 weeks after the cell injections and subjected to histological analysis and H&E staining. (**A)** Magnification of the NP area. **(B)** The injected iNCs were detected using immunofluorescence staining against Brachyury, Keratins 8, 18, and 19, and CTGF, and subjected to confocal microscopy. Colocalization of the notochordal markers and DiI-labeled iNCs or the DiO-labeled BM-MSCs in the NP area *in vivo* can be observed. **(C)** Gene expression analysis of the harvested IVDs at Week 12 after induction of degeneration and Week 8 after the cell/hydrogel injections shows expression of the human notochordal markers (Brachyury and Noto) in the NP and endplate areas, and expression of the NP differentiation markers GDF6 and Sox9 in the NP area. Values are expressed as means ± standard errors (bars) and are relative to the hydrogel only group; n=3; *p<0.05; ***p<0.001; ****p<0.000. NP - nucleus pulposus, AF - annulus fibrosus, EP - Endplate.

Encouraged by the *in vitro* results, we tested the sustainability of the iNC phenotype *in vivo* in a degenerated disc of a large animal (Fig. 6A). Minipigs underwent surgery during which the AFs of three IVDs (L1-2, L2-3, and L3-4) were punctured to induce degeneration (Fig. 6B). The progress of IVD degeneration was monitored using MRI as previously reported 62. Extensive degeneration was observed after 4 weeks (Fig. 6C); at this point, Geltrex hydrogel alone or mixed with iNCs or MSCs was injected into the degenerated discs, where the mixture solidifies at 37ºC (Fig. 6D). The degenerative statuses of the IVDs were visualized by MRI. In addition, qCEST signals were acquired from the degenerative IVDs. These signals had been correlated with pH levels inside the NP in our previous studies 47, 62. Quantitative qCEST analysis shows that pH levels in the NP of IVDs injected with hydrogel alone changed drastically 8 weeks post-injection, whereas no changes in pH levels in native IVDs (L4-5) were seen. A statistical analysis (ANOVA) of the qCEST exchange rate at the last time point—Week 12—showed a significant difference between both native IVDs and iNC-injected IVDs when compared with hydrogel-injected IVDs. No significant difference was observed between IVDs that received injections of MSCs and hydrogel. This suggests that iNCs may play a protective role by preventing the matrix degradation that is correlated with low pH levels in the degenerated disc. The hydrogel-alone and hydrogel-plus-MSCs groups did not show this effect (Fig. 6E). In discs injected with only hydrogel and in discs injected with hydrogel with MSCs, histological analysis revealed an abnormal IVD and cell matrix structure following puncture, with extensive fibrosis and formation of cell clusters in the NP, which is typical of degenerated IVDs 63 (Fig. 7A). Presence of immune cells in those IVDs was observed, indicating ongoing inflammation processes even 12 weeks after the injury. Although there was still matrix degradation in the iNC-injected IVD group, the NP structure was not as disturbed (Fig. 7A). Injected iNCs labeled with DiI lipophilic dye were detected in the NP region of the degenerated discs after 8 weeks *in vivo*, and immunofluorescence stains revealed that their phenotype was consistent with that found in the *in vitro* study—namely, they still expressed the notochordal markers Keratin 18, Keratin 19, Noto, and Brachyury (Fig. 7B). Gene expression analysis performed on tissue extracted from the treated IVDs also showed significant upregulation of the notochordal markers Brachyury and Noto in iNC-injected IVDs, specifically in the NP and endplate regions of the disc. Surprisingly, these markers were also detected in the end plate (EP) regions of the IVD, most probably due to random distribution and attachment of the injected cells to the endplates during the injection. Interestingly, the NP markers GDF6 and Sox9 were also upregulated in the NP region of the disc (Fig. 7C).

## Discussion

The main finding of this study is the development of new reproducible differentiation protocol for iPSC-derived notochordal cells that can maintain their phenotype *in vivo* and *in vitro* for at least 8 weeks and have protective effect of IVD in an injury-induced large animal degeneration model. Moreover, the injected iNCs could shift the intradiscal pH in the degenerated IVD.

One of the challenges facing IVD regeneration is the lack of an appropriate cell source—one that can be compatible with the harsh environment of the IVD yet safe and sufficiently available. NP cells have been isolated and characterized by multiple groups 64-66. Although characterization of the markers that identify their phenotype is still under debate 64, 67, there is no doubt that NPs are not easily obtained, of low expansion capacity and thus not too attractive for cell therapy. Our previous studies showed that both healthy and degenerated NP environments include stem cells or NP progenitor cells that could potentially be activated to induce regeneration to some extent 26, 68. Therefore, an alternative approach will be to enrich the cellular component of degenerating discs and reactivate the progenitor population of the disc.

The primary cause of disc degeneration remains unclear; however, there are several indications of a correlation between disc degeneration and the disappearance of NCs. Being this the case, NP tissue renewal in the IVD depends on the ability of notochordal progenitors to commit to the NP lineage and undergo terminal differentiation 69. Besides direct differentiation into NP cells, NCs have been shown to exert a protective paracrine effect on NP cells *in vitro* and *in vivo* 30, 32, 70. Therefore, restoration of disc NC function and prevention of degeneration remain the ultimate goal of current IVD research. In this study, we show a way to generate functioning notochordal cells from pluripotent cells.

Our 3-step differentiation protocol (Diagram 1) was conceived to replicate the embryogenic pathway from pluripotent to primitive streak mesoderm to notochord by using key small molecules, specific culture conditions, and a main transcription factor, Brachyury, which has been shown to regulate notochord development 71 while playing a pivotal role in IVD homeostasis 72. Our results show that short-term treatment of iPSCs with GSK3i leads to rapid differentiation: on Day 3 the cells reached the state of primitive streak mesoderm (Fig. 1). As in human development, this stage is transient: further culture of those cells in the presence of GSK3i leads to sporadic differentiation and massive cell death (Fig. 1). The notochord progenitor stage is also transient: it comprises a short time when the cells are in two-dimensional (2D) culture conditions (Fig. 2). Following Brachyury overexpression, these cells' phenotype decreased after Day 2 in culture. Conversely, when cultured in 3D hydrogels and hypoxic conditions, the phenotype remained consistent for at least 8 weeks both *in vitro* and *in vivo* (Figs. 3 & 6). GSK3i treatment at Step 1 induced Brachyury overexpression (Fig. 1C); however, further culture of the PSM cells in 3D hydrogels did not result in elevation of Brachyury unless it was overexpressed by non-viral transfection (Fig 3B). When PSM cells were also cultured in 3D hypoxic environment, none of the notochordal cell markers were upregulated in the 8 weeks of the experiment; increased expression was only observed when Brachyury was overexpressed (PSM-Br cells). Using parallel transfection with GFP encoding plasmid we have seen transfection efficiency of the PSM cells was around 70-80% (data not shown), therefore we need to assume that the iNC population is mixed with small proportion of PSM cells. Therefore, we consider sorting the pure population in our future studies. In this study, however, we were able to show that the mixed population showed notochordal phenotype after 8 weeks in 3D *in vitro* or *in vivo*, so we can speculate that the undifferentiated cells died off, similar to the 2D *in vitro* study and thus, relatively, enriching the iNC population. Alternatively, it may be assumed that a paracrine effect of the iNCs on PSM during the long term co culture was the cause that non-transfected cells differentiated to iNCs, similar to the MSCs that were cocultured with iNCs.

Brachyury is expressed in the IVD when the notochordal cells are present in the early childhood. It was reported to be associated with chondroma, serving as marker for chordoma cells, along with other notochordal markers like cytokeratins and CD24 73, while others reported a poor correlation between expression of Brachyury and chordomas 74. We have followed up the cells' gene expression for 8 weeks and found steady expression, consistent with observations in human notochordal cells. A longer follow up time of the cells' fate is in our high interest in future studies.

Induced pluripotent stem cells provide a unique opportunity to generate cells that are almost impossible to obtain from other sources. However, such cells often raise concerns related to the safety of their use in patients. In our study, we tested the PSM cells and iNCs *in vivo* for teratoma formation and any tumor was detected after 6 months *in vivo*. This test was performed in NOD/SCID mice using intramuscular injection; hence the cells had the optimal conditions of vascularized and nutrition-rich tissue such as skeletal muscle to support tumor formation. Our therapeutic approach, injection of cells into the IVD, will subject cells to a much harsher, hypoxic environment. Also, after injection of iNCs into porcine IVDs, no tumor was detected. Another safety concern is the development of a host-versus-graft inflammatory response against allogeneic iPSCs; the IVD is considered an immunoprivileged organ and thus can be treated with allogeneic or even xenogeneic human cells with a low risk of rejection 75. Likewise, allogeneic iPSCs do not induce a robust immunogenic response in other organs that lack functional antigen-presenting cells at the transplantation site, such as the kidney capsule 76 or cartilage 77. Next, future efforts will be devoted to establishing a genetically stable iPSC cell bank, and the iPSC line with the best match to the patient's HLA will be selected for cell therapy. Additionally, several treatments have been shown to reduce immunogenicity in iPSC-derived tissues 77, 78. Genomic instability and the consequent risk of tumorigenesis in iPSCs is another challenge for iPSC-derived therapy; however, once differentiated, the cells are more stable and can be subjected to quality control tests before implantation.

Although challenges may lie ahead, iPSC therapy holds great potential for the IVD degeneration field. It provides a source for autocrine or paracrine therapy. Our data indicate that iNCs exert a paracrine effect (Figs. 3-5). These cells exert a direct effect on promoting NP differentiation of MSCs in co-culture (Fig. 3, 4) as well as an indirect effect via conditioned media (Fig. 5). This feature of NCs was previously demonstrated in multiple experimental systems 32, 57-59, 79. Therefore, it can be defined as a function of NCs. In addition to the notochordal marker expression observed in iNCs *in vitro* and *in vivo* (Fig. 3, 7), the functional assay described in Fig. 5 reinforced the suggested phenotype of iNCs as notochordal cells. Theoretically, in the co-culture experiment the iNC could be affected by MSCs and thus the expression of notochordal markers increased overtime (Fig. 3). There are reports that show paracrine effect of MSCs in promoting cartilage formation by articular chondrocytes when co-cultured with MSCs 80. However, the data shown in Figure 5 indicate that iNC-conditioned media had direct effect on MSCs, strengthening the conclusion that the MSCs were affected by iNCs and not the opposite.

The ultimate function of iNCs is to regenerate the degenerated IVD *in vivo*. We have performed the first pilot study in which iNCs were injected into IVDs 4 weeks after induction of degeneration by annular puncture. This model mimics chronic age-related IVD degeneration. It is a powerful tool that can be used to induce the collapse of the extracellular matrix and, consequently, the degradation of the cellular component, thus demonstrating a tissue structure typical to degenerated IVDs 81-83. We haven't repeated the “injury only group” in this study, because the state of the degeneration of the disc can be appreciated in our previous publications 26, 27. Since the focus of this study was the effect of the iNCs on degenerated discs and the survival of the cells in the hostile environment of degenerated disc, our negative control group contained discs treated with hydrogel only. Our results show the initial process of regeneration that was reflected in the higher pH levels of discs treated with iNCs, compared to control discs and even to discs treated with MSCs (Fig. 6). Also, the histological analysis of discs postharvest shows more severe degeneration in control groups compared to iNC-treated discs (Fig. 7). To achieve substantial disc height or water content following treatment, further cell delivery optimization may be required. This will direct the application of the cells to the NP region and prevent leakage of the injected cells to the EP and AF areas. We speculate that the optimal treatment to ameliorate a degenerating disc will not be composed of only one type of cell but by a combination of cells—for example, adding MSCs and an adequate biomaterial to support differentiation of these cells—and will provide the biomechanical properties required to promote significant regeneration of the IVD. Nevertheless, the main finding of this study is the functionality of the iNC in the challenging environment of the collapsing and degenerating IVDs. It will be hard to argue that we recreated notochordal cells, since the direct comparison to human notochordal cells is practically impossible and because the environment of the developing notochord is quite different from the degenerating one. Furthermore, the developmental process of cell reprograming, and differentiation *in vitro* will never be identical to the *in vivo* development. However, since we can show functional protective and/or regenerative effect of the injected cells without causing side effects or introducing safety concerns, this is the most important outcome that has real potential as the next cell therapy for the IVD degeneration.

The previous studies, that have shown the feasibility of differentiating iPSCs to NP-like cells, mainly targeted a differentiated NP cell phenotype 37, 39, 40. In this study we are trying to bring the cells to the developmental stage before the upstream to the differentiated NP cell phenotype. The rationale is to generate progenitors and regulatory cells that can induce the resident cells to regenerate the IVD and in the future give rise to multiple IVD cells the will respond to the environmental ques and replace the lost NP cells and matrix.

One major limitation of the study was the necessity to use 3 different biomaterials for different properties. The TF gels were shown to provide biomechanical properties similar to the one found in the NP and promote differentiation 52. Most of the previous studies looking at the NCCM were performed in alginate beads 26, 84-86, that provide the 3D environment, however afford a relatively high surface/volume ratio and allow good factor exchange. Consequently, we used this well-establish system to test the hypothesis that our iNCs have paracrine effect on MSCs. Concomitantly, both biomaterials are not suitable for *in vivo* injection using minimally invasive technique. Alginate beads has to be formed in CaCl_2_ solution and TF gels are crosslinked under UV. Both systems are currently challenging for a use in an *in vivo* setting. Therefore, we used Geltrex^TM^ that undergoes gelation in 37ºC temperature, being easily injectable at room temp and coagulated intradiscal after injection. However, this material was not reported to support NC or NP differentiation. We are happy to report that despite that, iNCs maintained their phenotype and contributed to prevention of changes in the pH associated with degeneration, but further investigation is required in order to optimize the delivery system. In the future studies we plan to optimize the biomaterials that will both injectable and will provide the necessary biomechanical support and differentiation cues for the cells to induce de novo matrix formation.

## Methods

### Ethics Statement

The Cedars-Sinai IACUC and IRB committees approved all procedures described in this study.

### iPSC reprogramming

Healthy control dermal fibroblasts from one donor were obtained from the Coriell Institute for Medical Research (Camden, NJ) and additional dermal fibroblasts and blood T-cells were derived from two healthy donors at Cedars-Sinai Medical Center. Reprogramming of the cell lines was performed using plasmid vectors (Addgene, Cambridge, MA) and modification of a previously published protocol 87. A Human Dermal Fibroblast Nucleofection Kit (Lonza, Walkersville, MD) was used to make the virus-free iPSC lines. Briefly, fibroblasts (0.8 × 10^6^ cells/nucleofection) were harvested and centrifuged at 200*g* for 5 min. The cell pellet was resuspended carefully in Nucleofector Solution (VPD-1001, Lonza) and combined with the episomal plasmid expression of six factors—*OCT4*, *SOX2*, *KLF4*, *L-MYC*, *LIN28*, and *p53* shRNA—by plasmid nucleofection. This method has a significant advantage over viral transduction, because the genes do not integrate and are instead expressed episomally in a transient fashion. The cell/DNA suspension was transferred into the Nucleofection solution (Lonza), and a fibroblast-specific program was applied. All cultures were maintained under normal oxygen conditions (5% O_2_) during reprogramming, which further enhanced the efficiency of iPSC generation. The culture medium was maintained for 48 hrs. and then changed to human iPSC medium containing small molecules to enhance reprogramming efficiency. These small molecules included the following: (i) sodium butyrate; (ii) a glycogen synthase kinase 3β inhibitor of the Wnt/β-catenin signaling pathway (CHIR99021, Millipore, Temecula, CA); (iii) a mitogen-activated protein kinase pathway inhibitor; and (iv) a selective inhibitor of transforming growth factor-β type I receptor ALK5 kinase, type I activin/nodal receptor ALK4, and type I nodal receptor ALK7. Colonies with an embryonic stem/iPSC-like morphology appeared 25 to 31 days later. Subsequently, colonies displaying the best morphology were picked and transferred to layers of a standard hiPSC medium-and-Matrigel^TM^ matrix (BD Biosciences, Pharmingen, CA) for feeder-independent maintenance of hiPSCs in chemically defined mTeSR1 medium (Stem Cell Technologies, Vancouver, British Columbia, Canada). Three independent iPSC clones were picked from each reprogrammed fibroblast sample, further expanded, and cryopreserved. All the *in vitro* experiments were repeated with two additional iPS lines, one reprogrammed from dermal fibroblasts and one from blood samples of healthy volunteers.

### iNC differentiation

The derivation of iPSC-derived notochordal cells (iNCs) from iPSCs was performed using our 3-step protocol. During Step 1, the iPSCs were differentiated into Primitive Streak Mesoderm (PSM) cells via a 3-day exposure to 5µM GSK3 inhibitor (Millipore, Billerica, MA) in a manner similarly used in previously published studies 88, 89. The media was replaced every 24 hours supplemented with fresh 5µM GSK3 reconstituted in Dimethyl sulfoxide (DMSO). During Step 2, the GSK3i-treated cells were transfected using commercially available Nucleofection technology (Lonza, Basel, Switzerland) with human Brachyury-encoding pCMV6-AC-GFP vector plasmid (OriGene, Rockville, MD) and cultured for 2 days in Advanced-RPMI medium. The transfection efficiency was validated using flow cytometry to GFP+ cells, and transfection efficiency over 70% was considered successful. During Step 3, the cells were encapsulated in TF hydrogel as previously described 52, grown in hypoxic conditions (2% O_2_) for maturation *in vitro*, and harvested for RNA isolation and gene expression analysis or for vibro-sectioning and immunofluorescence staining.

### Conditioned medium test

Notochordal conditioned medium (NCCM) including NCCM derived from porcine IVDs was previously shown to have an effect on MSCs and to promote differentiation into an NP fate 57-59. To investigate the effect of the notochordal conditioned medium generated by iNCs *in vitro* on BM-MSCs, we encapsulated the iNCs in alginate beads, as previously reported 26, 84-86, and cultured them in NP media in hypoxic conditions (2% O_2_) 37. The conditioned medium was collected every 3 days, frozen in -80ºC and replaced with fresh media. As a positive control for NCCM, porcine NCs were isolated from freshly harvested porcine IVDs, as previously reported 57, 59. Human BM-MSCs were isolated from bone marrow aspirate, which was acquired from Lonza (Basel, Switzerland), by using a standard procedure 49, 55, 90. The BM-MSCs were expanded in culture and encapsulated in alginate beads using the same protocol and culture conditions as used with the iNCs. To test the functionality of iNCs to affect MSCs and enhance their differentiation into the NP phenotype, we encapsulated iNCs in alginate beads and cultured them in hypoxic conditions for 7 days. BM-MSCs were encapsulated in alginate beads in the same manner and cultured in the 50% fresh and 50% conditioned medium that was changed every 2 or 3 days. As a positive control, NCs were isolated from freshly harvested porcine NPs and separated using a 70-µm mesh strainer, as previously reported 57, 59. Porcine NCs were encapsulated using the same method, and porcine NC-conditioned medium (pNC-CM) was produced and applied to the BM-MSCs (Fig. 5A).

### Gene expression analysis

A quantitative RT-PCR was conducted on cells *in vitro*, TF hydrogel constructs, alginate beads, and IVDs harvested 12 weeks after degeneration (9 weeks after cell injection). The expression of genes from degenerative IVDs was compared to that of healthy IVDs. Total RNA was extracted from the AF and the NP by using the RNeasy Mini kit (Qiagen GmbH, Hilden, Germany) according to the manufacturer's protocol. RNA was retrotranscribed using random primers and reverse transcriptase (Promega Corp., Madison, WI, USA). Quantitative real-time PCR was performed with the aid of the ABI 7500 Prism system (Applied Biosystems, Foster City, CA). *18S* was used as a housekeeping gene control. Gene expression was quantified as RQ, relative fold increase comparing to the control group in each experiment. In the differentiation Step 1, undifferentiated iPSCs served as the calibrator. In Step 2, the PSM Day 3 cells served as the calibrator sample. In Step 3 (TF constructs) PSM-Br Day 2 cells served as the calibrator.

### Immunocytofluorescence (ICF)

The cells were plated on coverslips, fixed with 4% paraformaldehyde at room temperature for 15 min, washed with PBS and stored at 4ºC. For immunostaining, the cells were first permeabilized with 0.3% Triton in PBS for 1 hr and afterwards incubated with blocking solution (0.3% Triton + 5% donkey serum in PBS) for 1 hr in room temperature. Then the cells were stained with the primary antibodies listed in Supplemental Table 1 at 4ºC overnight or at room temperature for 3 hours. Secondary antibodies conjugated with a fluorochrome (Supplemental Table 1, Jackson ImmunoResearch Laboratories) were added, and coverslips were incubated for 2 hrs at room temperature. The cells were mounted using ProLong® Gold with DAPI (Molecular Probes, Life Technologies). Images were acquired using a 4-channel Laser Scanning Microscope 780 (Zeiss, Pleasanton, CA) with 20× magnification, z-stacking, and tile scanning. For zoom-in images, a single z-stacked image was generated using 40× magnification. All samples were scanned using the same gain and exposure settings.

### Flow cytometry

To verify the homogeneity of the mesodermal differentiation expression of CXCR4 and lack of expression of Sox17 was evaluated using flow cytometry. Anti-hCXCR4 488 Conjugated (Cat #FAB173G, R&D systems) and Anti-hSOX17 Allophycocyanin conjugated (Cat# IC1924A, R&D systems) were used following the manufacturer's protocol. In order to quantify the proportion of the survived cells in TF gels, gels were digested with collagenase as previously described 56, the cells were recovered, counted, their viability assessed and analyzed using flow cytometry using the far red and green filters to detect DiI and DiO labeled cells.

### iNC maturation in TF gel

The cells were dispersed in a TF hydrogel precursor solution, encapsulated after gelation, and subcultured within 3D TF hydrogels for up to 8 weeks, similar to a previously described method 52. Briefly, iNCs, MSCs, or iNCs plus MSCs were seeded in 1-kPa hydrogels containing 0.1% w/v Irgacure 2959 initiator (Ciba) at a seeding density of 3×10^6^ cells/ml. Fresh hydrogel precursors were prepared for each experiment. Each TF hydrogel (total volume 150 µl) was dispensed in a 5-mm-diameter silicone mold. The hydrogels were incubated for 5 min at 4ºC and then cross-linked under long-wave UV light (365 nm, 4e5 mW/cm^2^, Vilber Lourmat, Marne la Vallee, France) for 8 min. The cross-linked cell-seeded hydrogels were incubated in a humidified incubator (37ºC, 5% CO_2_) in culture medium suitable for NP tissue culture, as previously described 37. Cell culture medium was changed every 2 or 3 days throughout the experiment. Maturation and the NC phenotype were assessed using gene expression analysis and immunofluorescence stains. The TF gels were harvested at Week 8 and fixed in a 4% paraformaldehyde mixture (in phosphate-buffered saline, PBS) for 12 to 15 hours at 4ºC. The gels were mounted using super glue and were cut coronally into 50- to 70-µm sections using a vibratome. The sections were stained using the same protocol used for cells grown *in vitro*.

### Porcine IVD degeneration and cell injection

Three healthy female Yucatan miniature pigs with an average age of 10 months and weights ranging from ~40 to 60 kg were used in this study. Female pigs were used since females have been shown to have the higher predisposition to disc degeneration 91. While the animals were in a state of general anesthesia, NP degeneration was induced via a posterolateral approach at targeted levels (L1-2, L2-3, and L3-4). Under fluoroscopic guidance, a superficial 4-mm-deep stab incision was made, using a spine needle (18G), through the AF into the center of the NP and parallel to the endplate as previously reported 92. Four weeks later, the animals were imaged with MRI (Siemens Medical Solutions USA, Inc., PA) to verify degeneration. After the degeneration was verified, aliquots of 10^6^ MSCs or iNCs were labeled with DiI lipophilic dye and washed twice in PBS. Then the cells were resuspended in 100 µl Geltrex™ (#A1569601 ThermoFisher, Waltham, MA) injectable hydrogel and injected into the center of the degenerated discs by using a spinal needle under fluoroscopic guidance. Geltrex™, LDEV-Free Reduced Growth Factor Basement Membrane Matrix is a soluble form of basement membrane extracted from murine Engelbreth-Holm-Swarm (EHS) tumors. The major components of Geltrex™ matrix include laminin, collagen IV, entactin, and heparin sulfate proteoglycans. Each degenerated disc was treated differently (see Fig. 5A). One served as a control and was injected with hydrogel alone (100 µl). The second disc was injected with 10^6^ iNCs that had been pre-labeled with CM-DiI fluorescent dye and encapsulated in a hydrogel. The third disc was injected with 10^6^ BM-MSCs that had been pre-labeled with CM-DiI.

### *In vivo* MRI

Imaging experiments were performed using a 3T clinical MRI scanner (Magnetom Verio; Siemens Healthcare, Erlangen, Germany). The animals were placed in the right decubitus position with body array coils centered on the posterior aspect spinous process. Throughout the imaging procedures, anesthesia was maintained with isoflurane (1%-3.5%). CEST MRI was performed using a two-dimensional reduced field of view TSE CEST sequence (TR/TE 1⁄4 10,500/ 10ms, two averages, single shot) 93. For each IVD, images were acquired in the axial plane with 3-mm slice thickness, 140 × 40-mm^2^ field of view, and 1.1 × 1.1-mm^2^ spatial resolution. The CEST saturation module consists of 39 Gaussian-shaped pulses, with a duration tp = 80 ms for each pulse and an interpulse delay td =80ms (duty cycle = 50%, total saturation duration Ts = 6240 ms) at saturation flip angle 900, 1500, 2100, and 3000 B1 amplitudes = flip angle/ (gtp) = 0.73, 1.22, 1.71, and 2.45 µT; the Z-spectrum was acquired with 10 different saturation frequencies at ±1.6, ±1.3, ±1.0, ±0.7, and ±0.4 ppm. The scan time of the CEST experiment for each IVD was approximately 40 min. The B_0_ field was corrected using a water saturation shift referencing (WASSR) map 94.

### Histology and Immunofluorescence (IF)

The harvested IVDs were fixed in 4% formalin for 72 hours, decalcified using 0.5M EDTA solution (pH 7.4), dehydrated, and embedded in paraffin. Standard H&E staining was performed on the paraffin-embedded tissue sections in a manner previously reported 95, 96. For immunofluorescence staining, tissues were deparaffinized, and the antigens were retrieved by incubation in preheated Target Retrieval Solution^TM^ (Dako, Carpinteria, CA) overnight in 4ºC. Nonspecific antigens were blocked by applying blocking serum-free solution (Dako). Slides were stained with primary antibodies, as detailed in Supplemental Table 1. The primary antibodies were applied to the slides, after which the slides were incubated at 4ºC overnight and washed using PBS; the slides were then incubated with secondary antibodies (Supplemental Table 1) for 1 hr. at room temperature, after which they were again washed (Supplemental Table 1) with PBS. Finally, the slides were stained with 4',6-diamidino-2-phenylindole dihydrochloride (DAPI, 1µg/ml) for 5 min in the dark, after which they were again washed 3 times with PBS. A VectaMount mounting medium (Vector Laboratories, Burlingame, CA) was applied to the tissue. The slides were imaged using a 4-channel Laser Scanning Microscope 780 (Zeiss, Pleasanton, CA) with 20× magnification, z-stacking, and 3 × 5 tile scanning. For zoom-in images, a single z-stacked image was generated using 40× magnification. All samples were scanned using the same gain and exposure settings.

### Statistical Analysis

GraphPad Prism 6.0b software (GraphPad Prism, San Diego, CA) was used to analyze the data. Results are presented as means ± standard errors. Longitudinal data analysis was conducted using a 1-way ANOVA or a 2-way ANOVA with repeated measures and the Bonferroni post-test. To assess significance, p < 0.05 was considered statistically significant.

## Supplementary Material

Supplementary figures and tables.
