# Supplementary material for: Human iPSCs can be differentiated into notochordal cells that reduce intervertebral disc degeneration in a porcine model
Source: Theranostics. 2019 Oct 12;9(25):7506–24. doi: 10.7150/thno.34898 (PMC6831475; doi:10.7150/thno.34898)
Supplement: Supplementary file 1 — Supplementary figures and tables. [file thnov09p7506s1.pdf]

Supplemental Material

Supplemental Table 1: Antibodies used in this study

Type	Antigen	Conjugation	Host	Reactivity	Company	Catalog #	Dilution
1°	Brachyury	-	Rabbit	Human, Mouse	abcam	ab18530	1:100
1°	Keratin 18	-	Mouse	Human	abcam	ab7797	1:100
1°	Aggrecan	-	Mouse	Human, Cow	abcam	ab3778	
1°	Galectin3	-	Mouse	Human	abcam	ab7278	1:250
1°	SOX9	-	Rabbit	Human	abcam	ab182579	
1°	Keratin 19	-	Mouse	Human	Dako	M0888	1:50
1°	FOXF1	-	Rabbit	Human	abcam	ab168383	1:100
1°	NOTO	-	Rabbit	Human	Novus biological	NBP1-91009	1:100
1°	CD24	-	Mouse	Human	abcam	ab134375	1:100
1°	Keratin 8	-	Rabbit	Human, Mouse, Rat	abcam	ab59400	1:500
1°	CNN2	-	Goat	Human	abcam	ab129331	1:100
1°	FOXA2	-	Goat	Human, Mouse	abcam	ab5074	1:50
1°	SHH	-	Goat	Human, Mouse	Novus biological	AF464	1:50
1°	BASP1	-	Rabbit	Human	Santa Cruz	SC-66994	1:100
1°	4-Oct	-	Mouse	Human	abcam	ab105931	1:200
1°	MIXL1	-	Rabbit	Human	Bioss	Bs-12350R	1:100
1°	SOX2	-	Goat	Human	abcam	ab110145	1:100
1°	CTGF	-	Mouse	Human	MyBiosource	MBS200132 1	1:10
2°	Donkey	Alexa 647 Donkey Anti-Goat	Goat	Alexa 647	Jackson Immunoresearch	705-605-003	1:1000
2°	Donkey	Alexa 488 Donkey Anti- Rabbit	Rabbit	Alexa 488	Jackson Immunoresearch	711-545-152	1:1000
2°	Donkey	Rhodamine Donkey Anti-Mouse	Mouse	Rhodamine	Jackson Immunoresearch	715-025-150	1:1000
